# CXCL10, SCGN, and H2BC5 as Potential Key Genes Regulated by HCV Infection

**DOI:** 10.3390/genes15121502

**Published:** 2024-11-22

**Authors:** Çiğdem Yıldırım, Fatih Yay, Ayfer İmre, Orçun Soysal, Hasan Çağrı Yıldırım

**Affiliations:** 1 Department of Infectious Diseases and Clinical Microbiology, Nigde Training and Research Hospital, 51100 Nigde, Turkey; ayfer.imre@saglik.gov.tr (A.İ.); orcun.soysal@saglik.gov.tr (O.S.); 2 Clinical Biochemistry Laboratory, Nigde Training and Research Hospital, 51100 Nigde, Turkey; fatih.yay@saglik.gov.tr; 3 Department of Medical Oncology, Nigde Training and Research Hospital, 51100 Nigde, Turkey; cagri.yildirim@hacettepe.edu.tr

**Keywords:** hepatitis C, gene regulations, *CXCL10*, *SCGN*, *H2BC5*

## Abstract

**Introduction:** Hepatitis C infections are the main causes of fatal clinical conditions such as cirrhosis and HCC development, and biomarkers are needed to predict the development of these complications. Therefore, it is important to first determine which genes are deregulated in HCV-cells compared to healthy individuals. In our study, we aimed to identify the genes that are commonly upregulated or downregulated in HCV-infected cells using two different databases. **Material and Method:** In this study, differentially expressed genes (DEGs) that were commonly upregulated or downregulated were identified using publicly available databases GSE66842 and GSE84587. Afterwards, the interactions of DEG products with each other and other proteins were examined using the STRING database. Enrichment analyses of DEGs were performed using the Enrichr-KG web tool including the Gene Ontology Biological Process, KEGG, Jensen_DISEASES and DisGeNET libraries. miRNAs targeting DEGs were detected using miRDB and TargetScanHuman8.0. **Results:** In HCV-infected cells, the *CXCL10* expression is increased in both databases, while the *SCGN* and *H2BC5* (*HIST1H2BD*) expression is decreased. No direct interaction was found among CXCL10, SCGN, H2BC5 in the top ten proteins. CXCL10 is a member of Hepatitis C and viral protein interactions with cytokine and cytokine receptor KEGG pathways. H2BC5 is a member of viral carcinogenesis KEGG pathways. Predicted overlapping miRNAs targeted by common DEGs were as follows: 59 were where *CXCL10* was the estimated target, 22 where *SCGN* was the estimated target and *29* where *H2BC5* (*HIST1H2BD*) was the estimated target. **Conclusions:** Our study identified genes that were upregulated or downregulated in HCV-infected cells in both databases and miRNAs associated with these genes, using two different databases. This study creates groundwork for future studies to investigate whether these genes can predict HCV prognosis and HCV-associated HCC development.

## 1. Background

Hepatitis C infections represent a significant public health issue that can lead to chronic hepatitis, cirrhosis, and hepatocellular carcinoma (HCC) [1]. It is estimated that approximately 71 million people worldwide are chronically infected with hepatitis C, with around 400,000 deaths annually attributable to complications associated with the virus [2,3]. While infection can be prevented with vaccination as the primary prophylaxis for Hepatitis A and Hepatitis B, unfortunately, a vaccine has not yet been developed for Hepatitis C. Prior to the era of direct-acting antivirals (DAAs), sustained virological response rates were below 10% with interferon treatments; however, with the introduction of DAAs, these rates exceed 95% in non-cirrhotic patients and range from 80% to 90% in cirrhotic patients [4,5,6]. Furthermore, DAAs have been shown to reduce the risk of mortality by approximately 50% and the incidence of HCC by about 35% in individuals infected with hepatitis [7].

The hepatotropism of HCV is partially attributed to its binding to various receptors [8]. Studies have demonstrated that there are significant alterations in gene expression levels in individuals infected with HCV [9,10]. It is important to identify genes whose expression levels change in the case of HCV infection in order to be able to search for screening and treatment targets based on these genes in the future. Expressed microRNAs (miRNAs) play a crucial role in the regulation and expression of these genes. The liver-specific miRNA-122 is involved in enhancing the replication, translation, and stability of the HCV genome [11]. The dysregulation of miR-122 has been associated with aggressive forms of HCC [12]. Viral infections such as HCV can cause the dysregulation of miRNAs, leading to complications, including HCC [13]. Additionally, miR-122 is thought to serve as a potential biomarker in the development of HCC. It has been shown that levels of miR-122-5p, miR-222-3p, miR-146-5p, miR-150-5p, miR-30C-5p, miR-378a-3p, and miR-20a-5p are elevated in HCV-infected individuals, with a subsequent decrease in these levels following DAA treatment [14]. Genes that exhibit changes in expression levels in patients infected with HCV, along with their targeting miRNAs, are promising candidates for screening tests related to the risk of developing HCC [15].

In our study, we utilized two bioinformatics databases, one comprising Huh7.5.1 cells and the other consisting of primary human hepatocytes, to identify genes exhibiting changes in expression levels as a result of HCV infection. We also aimed to determine the pathways in which these genes are enriched, the proteins with which their products are associated, and the miRNAs that target these genes.

## 2. Materials and Methods

### 2.1. Detection of Differentially Expressed Genes (DEGs)

The Gene Expression Omnibus (GEO) DataSets (https://www.ncbi.nlm.nih.gov/gds) accessed on 21 August 2024 were used in this study. The analyzed datasets were GSE66842 [16] using the GPL10558 Illumina HumanHT-12 V4.0, San Diego, CA 92121 USA expression beadchip platform and GSE84587 [17] using the GPL6244 [HuGene-1_0-st] Affymetrix Human Gene 1.0 ST Array [transcript (gene) version] platform. The GSE66842 dataset contains gene expression profiles of differentiated Huh7.5.1 cells infected with the HCV Jc1 clone. Only data from 3 infected and 3 mock (control) samples on the 10th day of postinfection were used. Eleven cell line samples from days 3 and 7 were not used. The GSE84587 dataset contained 2 naive and 2 HCV-infected primary hepatocytes samples with postinfection day 11 data. Since viral RNA can be detected in the culture medium 10 days after HCV infection and since the infection was observed to spread to more than 80% of the cells and reach the highest titers in 8–10 days, datasets with data on the 10th and 11th day postinfection were used in our study.

Analyses were performed with the GEO2R (https://www.ncbi.nlm.nih.gov/geo/geo2r/) web tool to identify differentially expressed genes (DEGs) in both datasets. In the background, it uses GEOquery [18] and limma [19] to identify DEGs in microarray data. In the current study, the adjusted *p* score was calculated using the Benjamini and Hochberg false discovery rate method for multiple testing corrections. The log_2_ fold change threshold value was set to 1. The adjusted *p* score significance level cut-off was left as 0.05 by default. DEGs with adjusted *p* < 0.05 and Log_2_(FC) < −1 were considered downregulated, and those with adjusted *p* < 0.05 and Log_2_(FC) > 1 were considered upregulated. Genes without gene.symbol were not included in further analysis. The PubChem/Gene Symbol to Gene ID Conversion Tool (https://pubchem.ncbi.nlm.nih.gov/upload/tools/) [20] was used to identify the IDs of DEGs (Homo sapiens taxonomy ID: 9606) in both datasets. Then, a Venn diagram (https://bioinformatics.psb.ugent.be/webtools/Venn/) was utilized to identify the common upregulated and downregulated DEG IDs. The web tool (https://www.ncbi.nlm.nih.gov/gene) was applied to detect the official gene symbols of common DEGs.

### 2.2. Protein–Protein Interaction Analysis

The STRING database (https://string-db.org/) was utilized to analyze the interactions of DEG products with each other and other proteins, if any [21].

### 2.3. Enrichment Analysis of DEGs

The web tool Enrichr-KG [22] (https://maayanlab.cloud/enrichr-kg) was used for DEG analysis in Gene Ontology (GO) [23], Kyoto Encyclopedia of Genes and Genomes (KEGG) [24], Jensen_DISEASES for disease-gene associations [25], and DisGeNET for the integration of data on disease-associated genes and variants [26]. All processes were set to top terms 20, and *p* < 0.05 was considered significant.

### 2.4. Identification of Potential miRNAs Predictively Targeting DEGs

miRDB [27] (https://mirdb.org/) and TargetScanHuman8.0 (https://www.targetscan.org/vert_80/) databases were used to identify potential miRNAs targeting DEGs. TargetScan searches for miRNA seed region matches with conserved 8mer, 7mer, and 6mer regions. Predictions were also ranked based on the weighted context++ score [28]. Targets were estimated using a machine learning method by using the RNA-seq profiling dataset study and CLIP-ligation data together in the miRDB database [27]. Then, the intersecting miRNAs in both databases were detected with the help of a Venn Diagram (https://bioinformatics.psb.ugent.be/webtools/Venn/). The Cytoscape v3.10.2 program was utilized to visualize the interactions [29].

## 3. Results

### 3.1. DEGs and Ovarlapping DEGs

**Figure 1:**
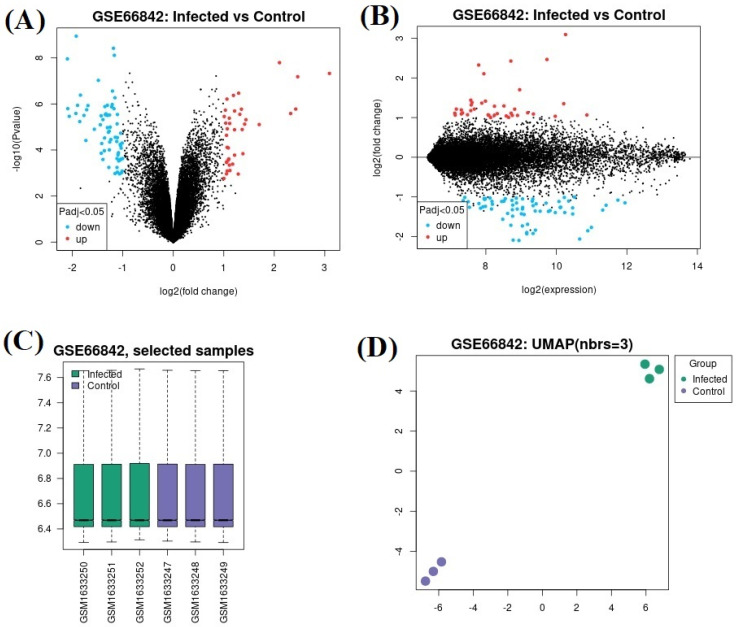
In GSE66842 dataset: (**A**) Volcano plot and (**B**) mean difference plot views of data distribution, (**C**) selected examples, and (**D**) UMAP plot views.

**Figure 2:**
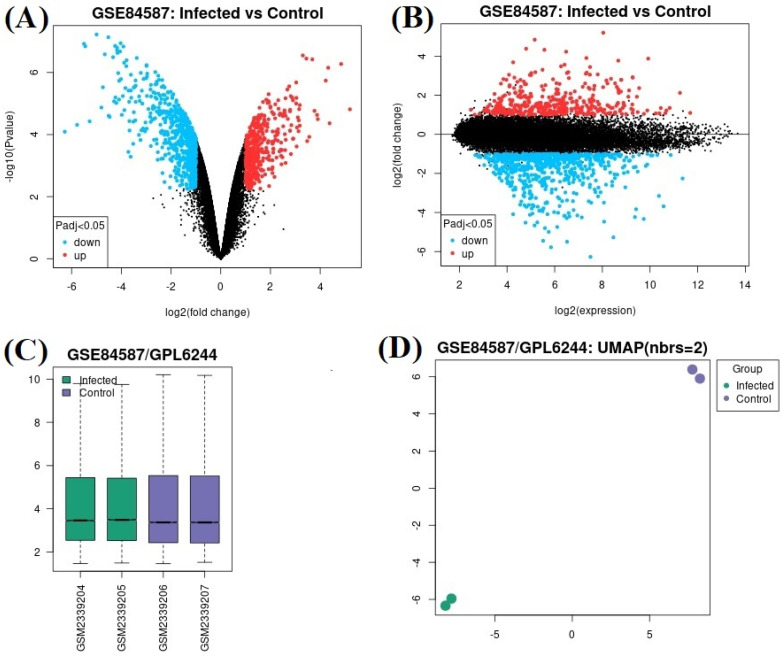
In GSE84587 dataset: (**A**) Volcano plot and (**B**) mean difference plot views of data distribution, (**C**) selected examples, and (**D**) UMAP plot views.

**Figure 3:**
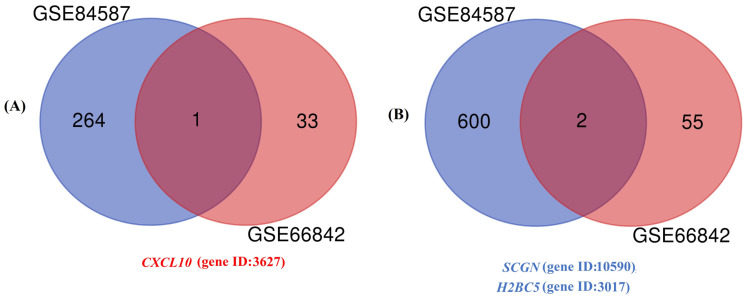
In the Venn diagram, common genes in GSE66842 and GSE84587 datasets: (**A**) upregulated and (**B**) downregulated.

In the GSE66842 datasetdataset, 34 genes were upregulated and 57 genes were downregulated (Figure 1A,B). In this data set, which samples are assigned to which group are shown in Figure 1C, and the sample numbers are shown in Figure 1D. In the GSE84587 dataset, 265 genes were upregulated and 602 genes were downregulated (Figure 2A,B). In this data set, which samples are assigned to which group are shown in Figure 2C, and the sample numbers are shown in Figure 2D. The only commonly upregulated gene was *CXCL10* with gene ID: 3627 (Figure 3A). The common downregulated genes were *SCGN* (gene ID: 10590), *H2BC5* (*HIST1H2BD*) (gene ID: 3017), respectively (Figure 3B). Genes showing separate and common upregulation in the datasets are listed in Supplemental Table S1, and genes showing separate and common downregulation are listed in Supplemental Table S2 according to their gene IDs.

### 3.2. Protein–Protein Interaction

**Figure 4:**
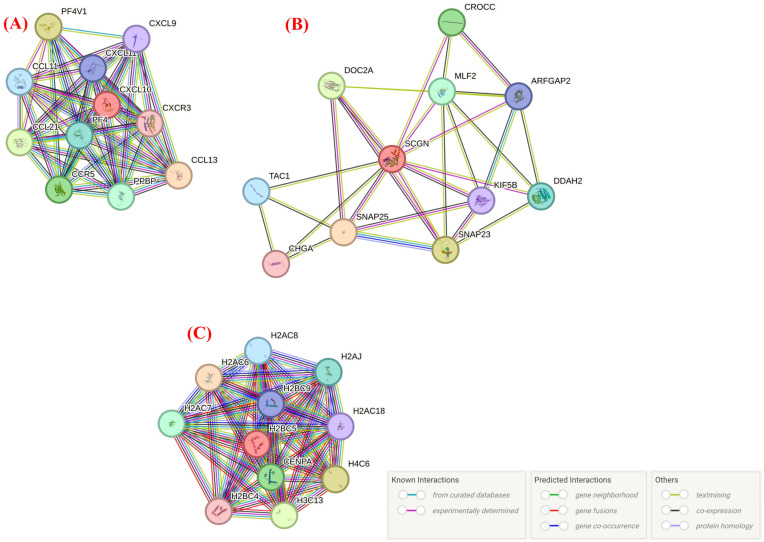
(**A**) Proteins that CXCL10 interacts with; (**B**) Proteins that SCGN interacts with; (**C**) Proteins that H2BC5 interacts with. Top 10 proteins with which CXCL10, SCGN, H2BC proteins interact the most: CXCL10, C-X-C motif chemokine 10; CCL13, C-C motif chemokine 13; PF4V1, Platelet factor 4 variant(4-74); CCL21, C-C motif chemokine 21; CCR5, C-C chemokine receptor type 5; PPBP, Connective tissue-activating peptide III(1-81); PF4, Platelet factor 4; CCL11, Eotaxin; CXCL11, C-X-C motif chemokine 11; CXCL9, C-X-C motif chemokine 9; CXCR3, C-X-C chemokine receptor type 3; [Isoform 1]; SCGN, Secretagogin, EF-hand calcium binding protein; SNAP25, Synaptosomal-associated protein 25; SNAP23, Synaptosomal-associated protein 23; DOC2A, Double C2-like domain-containing protein α; CROCC, Rootletin; MLF2, Myeloid leukemia factor 2; DDAH2, N(G),N(G)-dimethylarginine dimethylaminohydrolase 2; TAC1, C-terminal-flanking peptide; ARFGAP2, ADP-ribosylation factor GTPase-activating protein 2; KIF5B, Kinesin-1 heavy chain; CHGA, p-Glu serpinin precursor; H2BC5, Histone H2B type 1-D; H2AC6, Histone H2A type 1-C; H4C6, Histone H4; H3C13, Histone H3.2; CENPA, Histone H3-like centromeric protein A; H2AC7, Histone H2A type 1-D; H2AJ, Histone H2A.J; H2AC8, Histone H2A type 1-B/E; H2BC9, Histone H2B type 1-H; H2AC18, Histone H2A type 2-A; and H2BC4, Histone H2B type 1-C/E/F/G/I. Created using the STRING database (https://string-db.org/).

No direct interaction was found between CXCL10, SCGN, and H2BC5 (HIST1H2BD) in the top ten proteins. The top ten proteins that CXCL10 interacts with were as follows: C-C motif chemokine 13, Platelet factor 4 variant(4-74), C-C motif chemokine 21, C-C chemokine receptor type 5, Connective tissue-activating peptide III(1-81), Platelet factor 4, Eotaxin, C-X-C motif chemokine 11, C-X-C motif chemokine 9, C-X-C chemokine receptor type 3; [Isoform 1]. The interaction degrees are given in Table 1, and the interactions are visualized in Figure 4A.

The top ten proteins that SCGN interacts with were as follows: Synaptosomal-associated protein 25, Synaptosomal-associated protein 23, Double C2-like domain-containing protein α, Rootletin, Myeloid leukemia factor 2, N(G),N(G)-dimethylarginine dimethylaminohydrolase 2; C-terminal-flanking peptide; ADP-ribosylation factor GTPase-activating protein 2, Kinesin-1 heavy chain, and the p-Glu serpinin precursor. The interaction degrees are given in Table 2, and the interactions are visualized in Figure 4B.

The top ten proteins that H2BC5 interacts with were as follows: Histone H2A type 1-C, Histone H4, Histone H3.2, Histone H3-like centromeric protein A, Histone H2A type 1-D, Histone H2A.J, Histone H2A type 1-B/E, Histone H2B type 1-H, Histone H2A type 2-A, and Histone H2B type 1-C/E/F/G/I. The interaction degrees are given in Table 3, and the interactions are visualized in Figure 4C.

### 3.3. Pathways, Biological Processes, and Diseases in Which DEGs Are Enriched

**Figure 5:**
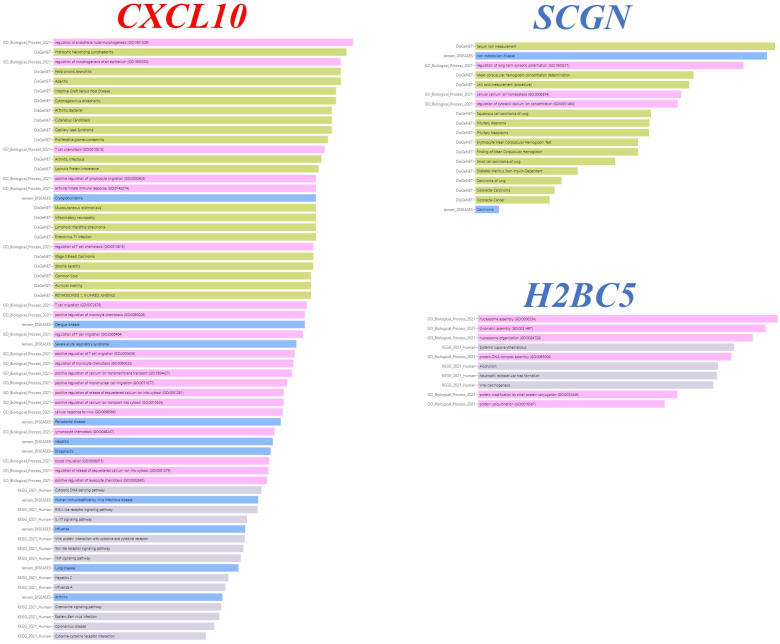
Bar charts of gene ontology (GO), Kyoto encyclopedia of genes and genomes (KEGG) pathway, Jensen_DISEASES, and DisGeNET analyses of CXCL10, SCGN, H2BC genes. Created using the web tool Enrichr-KG (https://maayanlab.cloud/enrichr-kg).

In terms of pathways that may be associated with HCV, the results were as follows: From KEGG: CXCL10 are members of the KEGG pathways as follows: Hepatitis C, the Cytokine–cytokine receptor interaction, Viral protein interaction with cytokine and a cytokine receptor, the TNF signaling pathway, the Toll-like receptor signaling pathway, the IL-17 signaling pathway, the RIG-I-like receptor signaling pathway, the Chemokine signaling pathway, and the Cytosolic DNA-sensing pathway. From Gene Ontology: CXCL10 belongs to the biological process as follows: the positive regulation of monocyte chemotaxis (GO:0090026), the regulation of monocyte chemotaxis (GO:0090025), the positive regulation of lymphocyte migration (GO:2000403), the regulation of T cell migration (GO:2000404), the positive regulation of T cell migration (GO:2000406), T cell chemotaxis (GO:0010818), the regulation of T cell chemotaxis (GO:0010819), T cell migration (GO:0072678), the positive regulation of mononuclear cell migration (GO:0071677), the positive regulation of leukocyte chemotaxis (GO:0002690), lymphocyte chemotaxis (GO:0048247), the cellular response to virus (GO:0098586), the antiviral innate immune response (GO:0140374), and the positive regulation of calcium ion transport into cytosol (GO:0010524). From Jensen lab: Arthritis, Cryoglobulinemia, and Hepatitis are associated with the gene CXCL10. From DisGeNET: Adenitis and Arthritis, Infectious, are associated with the gene CXCL10. All enrichments of CXCL10 are given in Table 4 with statistical significance values and visualized with bar charts in Figure 5.

**Table 4:** *CXCL10* enrichment analysis results.

Term	Library	*p*-Value	q-Value	z-Score	Combined Score
Cytosolic DNA-sensing pathway	KEGG_2021_Human	0.00315	0.0112	19,937	114,800
RIG-I-like receptor signaling pathway	KEGG_2021_Human	0.0035	0.0112	19,930	112,700
IL-17 signaling pathway	KEGG_2021_Human	0.0047	0.0112	19,906	106,700
Viral protein interaction with cytokine and cytokine receptor	KEGG_2021_Human	0.005	0.0112	19,900	105,400
Toll-like receptor signaling pathway	KEGG_2021_Human	0.0052	0.0112	19,896	104,600
TNF signaling pathway	KEGG_2021_Human	0.0056	0.0112	19,888	103,100
Hepatitis C	KEGG_2021_Human	0.00785	0.01212	19,843	96,180
Influenza A	KEGG_2021_Human	0.0086	0.01212	19,828	94,300
Chemokine signaling pathway	KEGG_2021_Human	0.0096	0.01212	19,808	92,030
Epstein–Barr virus infection	KEGG_2021_Human	0.0101	0.01212	19,798	90,980
Coronavirus disease	KEGG_2021_Human	0.0116	0.01265	19,768	88,100
Cytokine–cytokine receptor interaction	KEGG_2021_Human	0.01475	0.01475	19,705	83,090
Cryoglobulinemia	Jensen_DISEASES	0.0007	0.004	19,986	145,200
Dengue disease	Jensen_DISEASES	0.00095	0.004	19,981	139,000
Severe acute respiratory syndrome	Jensen_DISEASES	0.0012	0.004	19,976	134,300
Periodontal disease	Jensen_DISEASES	0.00185	0.004083	19,963	125,600
Hepatitis	Jensen_DISEASES	0.0023	0.004083	19,954	121,200
Encephalitis	Jensen_DISEASES	0.00245	0.004083	19,951	119,900
Human immunodeficiency virus infectious disease	Jensen_DISEASES	0.00345	0.004928	19,931	113,000
Influenza	Jensen_DISEASES	0.00495	0.006187	19,901	105,600
Lung disease	Jensen_DISEASES	0.00595	0.006611	19,881	101,900
Arthritis	Jensen_DISEASES	0.0093	0.0093	19,814	92,680
Regulation of endothelial tube morphogenesis (GO:1901509)	GO_Biological_Process_2021	0.00025	0.00525	19,995	165,800
Regulation of morphogenesis of an epithelium (GO:1905330)	GO_Biological_Process_2021	0.00035	0.00525	19,993	159,100
T cell chemotaxis (GO:0010818)	GO_Biological_Process_2021	0.00055	0.00525	19,989	150,000
Positive regulation of lymphocyte migration (GO:2000403)	GO_Biological_Process_2021	0.0007	0.00525	19,986	145,200
Antiviral innate immune response (GO:0140374)	GO_Biological_Process_2021	0.0007	0.00525	19,986	145,200
Regulation of T cell chemotaxis (GO:0010819)	GO_Biological_Process_2021	0.00075	0.00525	19,985	143,800
T cell migration (GO:0072678)	GO_Biological_Process_2021	0.0009	0.00525	19,982	140,100
Positive regulation of monocyte chemotaxis (GO:0090026)	GO_Biological_Process_2021	0.00095	0.00525	19,981	139,000
Regulation of T cell migration (GO:2000404)	GO_Biological_Process_2021	0.001	0.00525	19,980	138,000
Positive regulation of T cell migration (GO:2000406)	GO_Biological_Process_2021	0.00125	0.00525	19,975	133,500
Regulation of monocyte chemotaxis (GO:0090025)	GO_Biological_Process_2021	0.0013	0.00525	19,974	132,700
Positive regulation of calcium ion transmembrane transport (GO:1904427)	GO_Biological_Process_2021	0.00135	0.00525	19,973	132,000
Positive regulation of mononuclear cell migration (GO:0071677)	GO_Biological_Process_2021	0.00155	0.00525	19,969	129,200
Positive regulation of release of sequestered calcium ion into cytosol (GO:0051281)	GO_Biological_Process_2021	0.0017	0.00525	19,966	127,300
Positive regulation of calcium ion transport into cytosol (GO:0010524)	GO_Biological_Process_2021	0.0017	0.00525	19,966	127,300
Cellular response to virus (GO:0098586)	GO_Biological_Process_2021	0.00175	0.00525	19,965	126,700
Lymphocyte chemotaxis (GO:0048247)	GO_Biological_Process_2021	0.0022	0.006212	19,956	122,100
Blood circulation (GO:0008015)	GO_Biological_Process_2021	0.00255	0.006261	19,949	119,100
Regulation of release of sequestered calcium ion into cytosol (GO:0051279)	GO_Biological_Process_2021	0.0026	0.006261	19,948	118,700
Positive regulation of leukocyte chemotaxis (GO:0002690)	GO_Biological_Process_2021	0.0027	0.006261	19,946	118,000
Histiocytic Necrotizing Lymphadenitis	DisGeNET	0.0003	0.01002	19,994	162,200
Fetid chronic bronchitis	DisGeNET	0.00035	0.01002	19,993	159,100
Adenitis	DisGeNET	0.00035	0.01002	19,993	159,100
Intestinal Graft Versus Host Disease	DisGeNET	0.0004	0.01002	19,992	156,400
Cytomegalovirus encephalitis	DisGeNET	0.0004	0.01002	19,992	156,400
Arthritis, Bacterial	DisGeNET	0.00045	0.01002	19,991	154,100
Cutaneous Candidiasis	DisGeNET	0.00045	0.01002	19,991	154,100
Capillary Leak Syndrome	DisGeNET	0.00045	0.01002	19,991	154,100
Proliferative glomerulonephritis	DisGeNET	0.0005	0.01002	19,990	151,900
Arthritis, Infectious	DisGeNET	0.0006	0.01002	19,988	148,300
Lysinuric Protein Intolerance	DisGeNET	0.00065	0.01002	19,987	146,700
Mucocutaneous leishmaniasis	DisGeNET	0.0007	0.01002	19,986	145,200
Inflammatory neuropathy	DisGeNET	0.0007	0.01002	19,986	145,200
Lymphoid interstitial pneumonia	DisGeNET	0.0007	0.01002	19,986	145,200
Enterovirus 71 infection	DisGeNET	0.0007	0.01002	19,986	145,200
Stage 0 Breast Carcinoma	DisGeNET	0.00075	0.01002	19,985	143,800
Stromal keratitis	DisGeNET	0.00075	0.01002	19,985	143,800
Common Cold	DisGeNET	0.0008	0.01002	19,984	142,500
Auricular swelling	DisGeNET	0.0008	0.01002	19,984	142,500
RETINOSCHISIS 1, X-LINKED, JUVENILE	DisGeNET	0.0008	0.01002	19,984	142,500

From Jensen lab: Carcinoma is associated with the gene SCGN. All the enrichments belonging to SCGN are given in Table 5 with statistical significance values and visualized with bar charts in Figure 5.

**Table 5:** *SCGN* enrichment analysis results.

Term	Library	*p*-Value	q-Value	z-Score	Combined Score
Iron metabolism disease	Jensen_DISEASES	0.00085	0.0017	19,983	141,300
Carcinoma	Jensen_DISEASES	0.5659	0.5659	8682	4943
Regulation of long-term synaptic potentiation (GO:1900271)	GO_Biological_Process_2021	0.0015	0.0045	19,970	129,900
Cellular calcium ion homeostasis (GO:0006874)	GO_Biological_Process_2021	0.0068	0.0074	19,864	99,140
Regulation of cytosolic calcium ion concentration (GO:0051480)	GO_Biological_Process_2021	0.0074	0.0074	19,852	97,400
Serum iron measurement	DisGeNET	0.0007	0.0091	19,986	145,200
Mean corpuscular hemoglobin concentration determination	DisGeNET	0.00505	0.02427	19,899	105,200
Uric acid measurement (procedure)	DisGeNET	0.0056	0.02427	19,888	103,100
Squamous cell carcinoma of lung	DisGeNET	0.01415	0.03144	19,717	83,960
Pituitary Adenoma	DisGeNET	0.0147	0.03144	19,706	83,160
Pituitary Neoplasms	DisGeNET	0.0148	0.03144	19,704	83,020
Erythrocyte Mean Corpuscular Hemoglobin Test	DisGeNET	0.01935	0.03144	19,613	77,370
Finding of Mean Corpuscular Hemoglobin	DisGeNET	0.01935	0.03144	19,613	77,370
Small-cell carcinoma of lung	DisGeNET	0.03365	0.04861	19,327	65,550
Diabetes Mellitus, Non-Insulin-Dependent	DisGeNET	0.0836	0.1087	18,328	45,480
Carcinoma of lung	DisGeNET	0.1238	0.1463	17,524	36,610
Colorectal Carcinoma	DisGeNET	0.1465	0.1588	17,069	32,780
Colorectal Cancer	DisGeNET	0.1649	0.1649	16,702	30,100

H2BC5 is a member of the viral carcinogenesis KEGG pathway. All enrichments belonging to H2BC5 are given in Table 6 with statistical significance values and visualized with bar charts in Figure 5.

**Table 6:** *H2BC5* enrichment analysis results.

Term	Library	*p*-Value	q-Value	z-Score	Combined Score
Systemic lupus erythematosus	KEGG_2021_Human	0.00675	0.01015	19,865	99,290
Alcoholism	KEGG_2021_Human	0.0093	0.01015	19,814	92,680
Neutrophil extracellular trap formation	KEGG_2021_Human	0.00945	0.01015	19,811	92,350
Viral carcinogenesis	KEGG_2021_Human	0.01015	0.01015	19,797	90,870
Nucleosome assembly (GO:0006334)	GO_Biological_Process_2021	0.0029	0.0094	19,942	116,500
Chromatin assembly (GO:0031497)	GO_Biological_Process_2021	0.00365	0.0094	19,927	111,900
Nucleosome organization (GO:0034728)	GO_Biological_Process_2021	0.0047	0.0094	19,906	106,700
Protein-DNA complex assembly (GO:0065004)	GO_Biological_Process_2021	0.00715	0.01072	19,857	98,110
Protein modification by small protein conjugation (GO:0032446)	GO_Biological_Process_2021	0.02045	0.02454	19,591	76,200
Protein ubiquitination (GO:0016567)	GO_Biological_Process_2021	0.02625	0.02625	19,475	70,890

**Figure 6:**
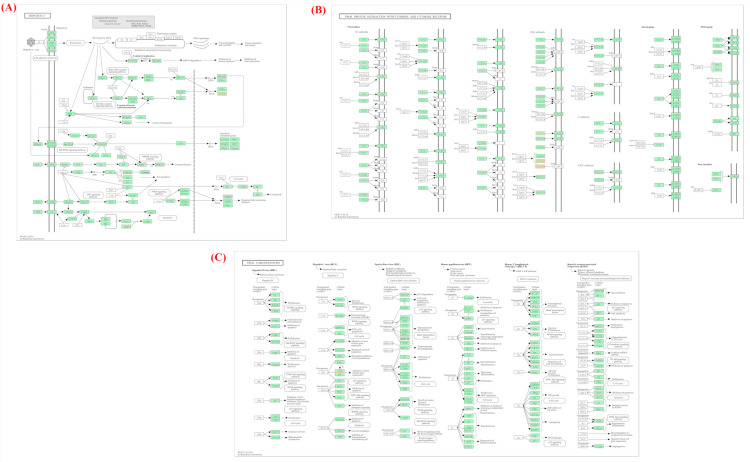
(**A**) CXCL10 in hepatitis C KEGG pathway, (**B**) CXCL10 in viral protein interaction with cytokine and cytokine receptor KEGG pathway; (**C**) H2BC5 in viral carcinogenesis KEGG pathway. Images from KEGG database (https://www.genome.jp/kegg/genes.html).

The Hepatitis C and Viral protein interaction with cytokine and cytokine receptor KEGG pathways, of which CXCL10 is a member, are shown in Figure 6A,B, and the viral carcinogenesis KEGG pathway, of which H2BC5 is a member, is shown in Figure 6C.

### 3.4. miRNAs Predictively Targeting DEGs

**Figure 7:**
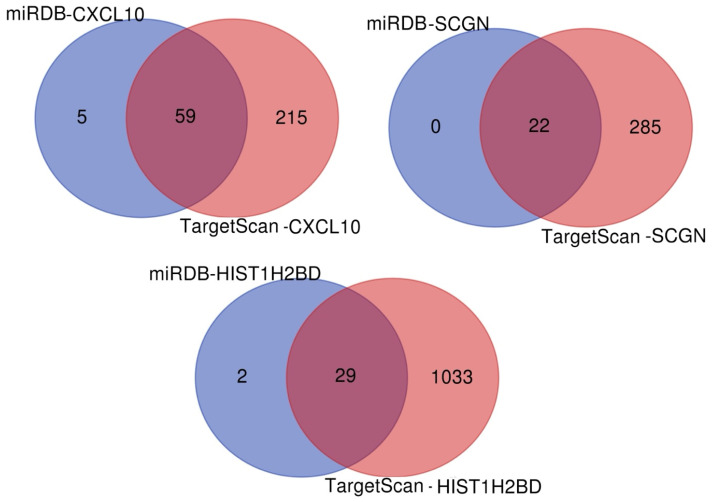
Separate and overlapping numbers of miRNAs that are potential targets of *CXCL10*, *SCGN*, and *H2BC5* (*HIST1H2BD*) according to miRDB and TargetScanHuman8.0.

**Figure 8:**
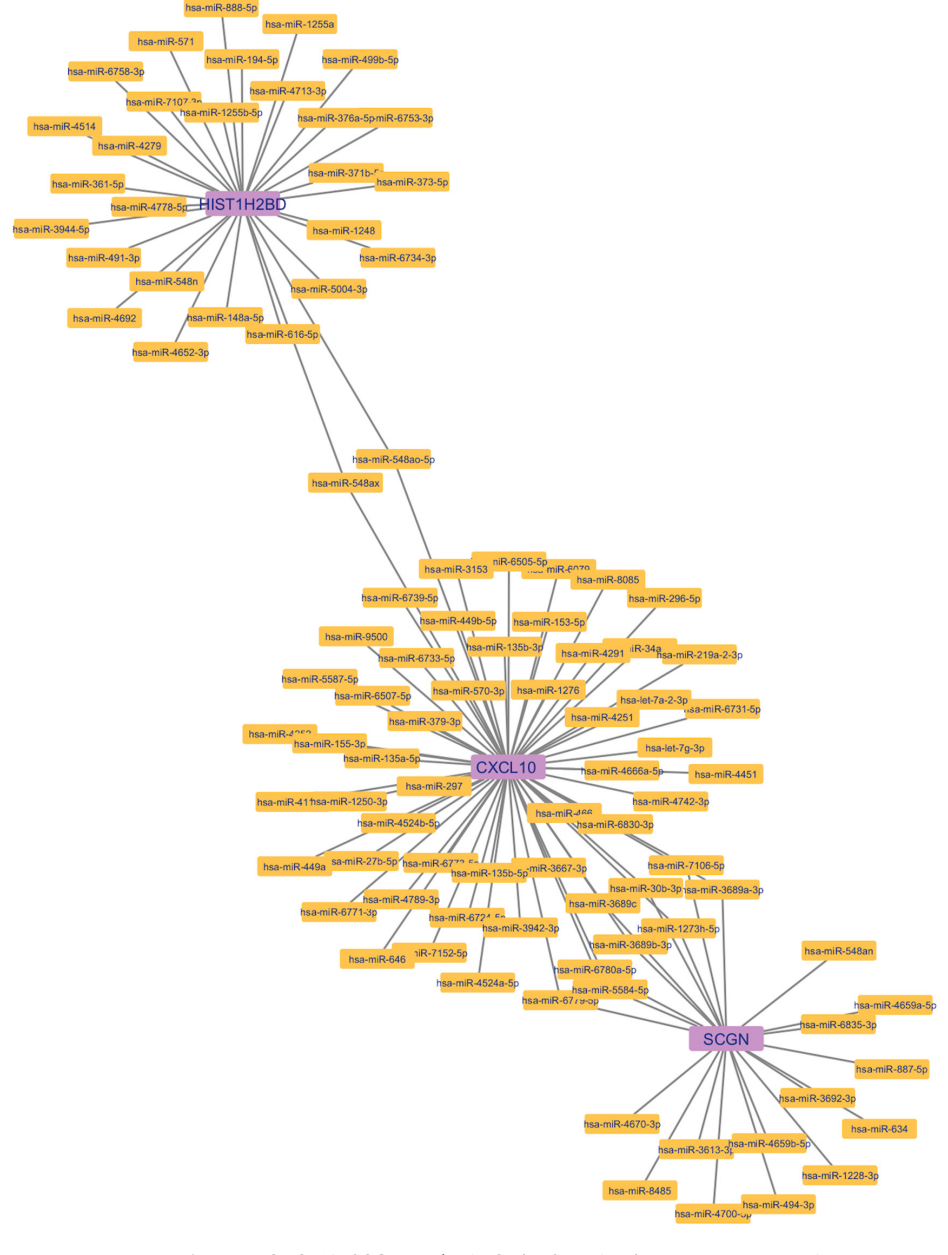
*CXCL10*, *SCGN*, and *H2BC5* (*HIST1H2BD*) target gene–miRNA interactions.

TargetScanHuman8.0 included CXCL10 ENST00000306602.1, Human HIST1H2BD ENST00000289316.2 transcripts. For SCGN, the Representative (most prevalent) transcript (ENSG00000079689.9) was used. According to the results obtained using the Venn diagram in the TargetScanHuman8.0 and miRDB databases, 59 overlapping miRNAs were detected, including *CXCL10* as a target, 22 *SCGN* as a target, and 29 *H2BC5* (*HIST1H2BD*) as a target (Figure 7 and Table 7). Of these, hsa-miR-548ao-5p and hsa-miR-548ax were found to target both *CXCL10* and *HIST1H2BD*. hsa-miR-3689c, hsa-miR-7106-5p, hsa-miR-1273h-5p, hsa-miR-30b-3p, hsa-miR-6780a-5p, hsa-miR-5584-5p, hsa-miR-3689b-3p, hsa-miR-3689a-3p, and hsa-miR-6779-5p were found to target both *CXCL10* and *SCGN*. Target miRNA interactions are visualized in Figure 8.

## 4. Discussion

Our study is the first to demonstrate the upregulation of *CXCL10* and downregulation of *SCGN* and *H2BC5* following HCV infection using two distinct databases.

Gene regulation is mediated by miRNAs, with over 1,000 miRNAs currently identified [30]. Gene analyses are conducted more accurately using real-time reverse transcription-PCR (RT-PCR). Changes in gene expression in patients infected with HCV affect transcriptional networks regulated by interferons (IFNs), including both IFNα/β-inducible genes (such as STAT1, STAT2, ISGF3G/IRF9, IFI27, G1P3, G1P2, OAS2, and MX1) and IFNγ-inducible genes (including CXCL9, CXCL10, and CXCL11) [9,31]. miRNAs are involved in regulating cellular differentiation, proliferation, and apoptosis. Previous studies have shown that miR-122 levels are inversely correlated with HCV replication and infectious viral production [11]. It was also demonstrated that IFNβ regulates the expression of numerous cellular miRNAs in vitro, and eight of these IFNβ-induced miRNAs have predicted targeting sites within the HCV genomic RNA [32]. Additionally, IFNβ leads to a significant decrease in miR-122 expression. These findings strongly support the notion that the IFN system utilizes cellular miRNAs to combat HCV infection.

CXCL10 (interferon-inducible protein-10, IP-10) binds to its receptor CXCR3, allowing it to attract CXCR3+ cells such as T lymphocytes, monocytes, and NK cells [33]. Numerous studies have associated CXCL10 expression with poor response to anti-HCV treatment and poor prognosis, as well as with HCV-related HCC [34,35,36]. The association of CXCL10 with CXCR3 increases tumor proliferation and migration and plays a role in the metastasis mechanism, so, in the future, CXCL10 can be used both in HCV-associated HCC screening, and there is a possibility that CXCL10-targeting therapies can be used in the treatment of HCV-associated HCC [37].

Secretagogin (SCGN) is an EF-hand calcium (Ca²^+^) binding protein that is highly expressed in pancreatic β cells [38]. Previous studies have indicated that SCGN plays a critical role in various aspects of pancreatic β cell function, including the regulation of insulin secretion, the proliferation of α and β cells, and the maintenance of β cell specification within islet cells [39,40]. To date, only one study has investigated the relationship between SCGN expression and HCV, which reported increased expression in individuals infected with HCV genotype 3a [41]. Our study is the first to show that SCGN expression is downregulated in both datasets containing HCV Jc1 clone-infected cells and HCV-infected primary hepatocytes.

Regarding H2BC5 (HIST1H2BD), there is limited information available. Bioinformatic analyses have shown that H2BC5 is more highly expressed in lung adenocarcinoma and squamous cell carcinoma tissues compared to healthy tissue, with high expression correlating with better survival in lung cancer patients [42]. Another study identified a relationship between H2BC5 expression and osimertinib resistance in patients undergoing NGS analysis [43]. However, there is no existing data on H2BC5 expression in HCV-infected cell lines. Our analysis revealed a decrease in H2BC5 expression in both databases concerning HCV-infected cell lines.

This study is significant for evaluating two different databases and identifying commonly upregulated or downregulated genes in both; however, we acknowledge certain limitations. The primary limitation is that our analysis was conducted using publicly available bioinformatic databases, which precludes an examination of the relationship between HCV and the potential development of HCC. Nonetheless, the upregulated and downregulated genes identified in our findings provide preliminary insights for future studies aimed at predicting HCC development in individuals infected with HCV. Future studies are needed to examine the relationship between changes in the levels of genes we detected during follow-up in HCV-infected individuals and the development of HCC.

## 5. Conclusions

miRNAs and gene expression changes are promising candidates for biomarkers in various diseases. In our study, we demonstrated alterations in the expression levels of CXCL10, SCGN, and H2BC5 in cells infected with HCV using two distinct databases. Identifying these genes and determining the associated miRNAs is crucial for future studies aimed at predicting the prognosis of HCV or identifying biomarkers that can predict the development of HCV-related HCC.

## Figures and Tables

**Table 1:** Top 10 proteins with which CXCL10 interacts functionally and physically.

Proteins that CXCL10 Interacts with	Combined Confidence of the Functional Interaction	Combined Confidence of the Physical (Co-Complex) Interaction
CCL13	0.999 (very high)	0.734 (high)
PF4V1	0.999 (very high)	0.972 (very high)
CCL21	0.999 (very high)	0.817 (high)
CCR5	0.999 (very high)	0.995 (very high)
PPBP	0.999 (very high)	0.834 (high)
PF4	0.999 (very high)	0.972 (very high)
CCL11	0.999 (very high)	0.747 (high)
CXCL11	0.999 (very high)	0.974 (very high)
CXCL9	0.999 (very high)	0.981 (very high)
CXCR3	0.999 (very high)	0.996 (very high)

CXCL10, C-X-C motif chemokine 10; CCL13, C-C motif chemokine 13; PF4V1, Platelet factor 4 variant(4-74); CCL21, C-C motif chemokine 21; CCR5, C-C chemokine receptor type 5; PPBP, Connective tissue-activating peptide III(1-81); PF4, Platelet factor 4; CCL11, Eotaxin; CXCL11, C-X-C motif chemokine 11; CXCL9, C-X-C motif chemokine 9; and CXCR3, C-X-C chemokine receptor type 3; [Isoform 1].

**Table 2:** Top 10 proteins with which SCGN interacts functionally and physically.

Proteins that SCGN Interacts with	Combined Confidence of the Functional Interaction	Combined Confidence of the Physical (Co-Complex) Interaction
SNAP25	0.897 (high)	0.483 (medium)
SNAP23	0.872 (high)	0.674 (medium)
DOC2A	0.846 (high)	0.658 (medium)
CROCC	0.832 (high)	0.587 (medium)
MLF2	0.726 (high)	0.469 (medium)
DDAH2	0.723 (high)	0.546 (medium)
TAC1	0.709 (high)	No evidence
ARFGAP2	0.687 (medium)	0.292 (exploratory)
KIF5B	0.636 (medium)	0.452 (medium)
CHGA	0.634 (medium)	0.245 (exploratory)

SCGN, Secretagogin, EF-hand calcium binding protein; SNAP25, Synaptosomal-associated protein 25; SNAP23, Synaptosomal-associated protein 23; DOC2A, Double C2-like domain-containing protein α; CROCC, Rootletin; MLF2, Myeloid leukemia factor 2; DDAH2, N(G),N(G)-dimethylarginine dimethylaminohydrolase 2; TAC1, C-terminal-flanking peptide; ARFGAP2, ADP-ribosylation factor GTPase-activating protein 2; KIF5B, Kinesin-1 heavy chain; and CHGA, p-Glu serpinin precursor.

**Table 3:** Top 10 proteins with which H2BC5 interacts functionally and physically.

Proteins that H2BC5 Interacts with	Combined Confidence of the Functional Interaction	Combined Confidence of the Physical (Co-Complex) Interaction
H2AC6	0.998 (very high)	0.848 (high)
H4C6	0.992 (very high)	0.953 (very high)
H3C13	0.985 (very high)	0.940 (very high)
CENPA	0.983 (very high)	0.861 (high)
H2AC7	0.983 (very high)	0.941 (very high)
H2AJ	0.979 (very high)	0.956 (very high)
H2AC8	0.977 (very high)	0.737 (high)
H2BC9	0.977 (very high)	0.887 (high)
H2AC18	0.975 (very high)	0.668 (medium)
H2BC4	0.972 (very high)	0.903 (very high)

H2BC5, Histone H2B type 1-D; H2AC6, Histone H2A type 1-C; H4C6, Histone H4; H3C13, Histone H3.2; CENPA, Histone H3-like centromeric protein A; H2AC7, Histone H2A type 1-D; H2AJ, Histone H2A.J; H2AC8, Histone H2A type 1-B/E; H2BC9, Histone H2B type 1-H; H2AC18, Histone H2A type 2-A; and H2BC4, Histone H2B type 1-C/E/F/G/I.

**Table 7:** Overlapping miRNAs in TargetScanHuman8.0 and miRDB databases where *CXCL10*, *SCGN*, and *H2BC5* (*HIST1H2BD*) are potential targets.

Targeted Genes	Total Count	Predicted miRNAs
*CXCL10*	59	hsa-miR-34c-5p hsa-miR-449b-5p hsa-miR-4789-3p hsa-miR-1276 hsa-miR-135b-5p hsa-let-7a-2-3p hsa-miR-4524a-5p hsa-miR-3689c hsa-miR-7106-5p hsa-miR-6739-5p hsa-miR-6771-3p hsa-miR-3667-3p hsa-miR-4742-3p hsa-miR-4524b-5p hsa-miR-6773-5p hsa-miR-449a hsa-miR-6733-5p hsa-miR-6505-5p hsa-miR-5584-5p hsa-miR-155-3p hsa-let-7g-3p hsa-miR-411-3p hsa-miR-27b-5p hsa-miR-5587-5p hsa-miR-6079 hsa-miR-548ax hsa-miR-34a-5p hsa-miR-466 hsa-miR-9500 hsa-miR-1273h-5p hsa-miR-6731-5p hsa-miR-30b-3p hsa-miR-135a-5p hsa-miR-297 hsa-miR-4291 hsa-miR-6830-3p hsa-miR-4451 hsa-miR-4251 hsa-miR-1250-3p hsa-miR-3689a-3p hsa-miR-379-3p hsa-miR-3153 hsa-miR-646 hsa-miR-6507-5p hsa-miR-3942-3p hsa-miR-570-3p hsa-miR-153-5p hsa-miR-135b-3p hsa-miR-7152-5p hsa-miR-548ao-5p hsa-miR-6780a-5p hsa-miR-6724-5p hsa-miR-296-5p hsa-miR-8085 hsa-miR-4252 hsa-miR-219a-2-3p hsa-miR-3689b-3p hsa-miR-4666a-5p hsa-miR-6779-5p
*SCGN*	22	hsa-miR-8485 hsa-miR-3689c hsa-miR-3613-3p hsa-miR-7106-5p hsa-miR-4659a-5p hsa-miR-494-3p hsa-miR-634 hsa-miR-4659b-5p hsa-miR-548an hsa-miR-4670-3p hsa-miR-5584-5p hsa-miR-4700-5p hsa-miR-1273h-5p hsa-miR-30b-3p hsa-miR-3692-3p hsa-miR-1228-3p hsa-miR-887-5p hsa-miR-3689a-3p hsa-miR-6835-3p hsa-miR-6780a-5p hsa-miR-3689b-3p hsa-miR-6779-5p
*HIST1H2BD*	29	hsa-miR-1248 hsa-miR-4652-3p hsa-miR-1255b-5p hsa-miR-361-5p hsa-miR-5004-3p hsa-miR-491-3p hsa-miR-571 hsa-miR-548ax hsa-miR-4514 hsa-miR-6734-3p hsa-miR-373-5p hsa-miR-499b-5p hsa-miR-888-5p hsa-miR-4778-5p hsa-miR-616-5p hsa-miR-548n hsa-miR-4713-3p hsa-miR-3944-5p hsa-miR-371b-5p hsa-miR-7107-3p hsa-miR-6753-3p hsa-miR-194-5p hsa-miR-148a-5p hsa-miR-1255a hsa-miR-6758-3p hsa-miR-4279 hsa-miR-376a-5p hsa-miR-4692 hsa-miR-548ao-5p

## Data Availability

The datasets generated and analyzed during the current study are available in The Gene Expression Omnibus (GEO) DataSets (https://www.ncbi.nlm.nih.gov/gds) GSE66842 and GSE84587, the STRING database (https://string-db.org/), Enrichr-KG (https://maayanlab.cloud/enrichr-kg), miRDB (https://mirdb.org/), TargetScanHuman8.0 (https://www.targetscan.org/vert_80/).

## Supplementary Materials

The following supporting information can be downloaded at https://www.mdpi.com/article/10.3390/genes15121502/s1, Table S1: Differentially upregulated genes in the datasets; Video S1: title; Table S2: Differentially downregulated genes in the datasets.
