# Supplementary material for: CXCL10, SCGN, and H2BC5 as Potential Key Genes Regulated by HCV Infection
Source: Genes (Basel). 2024 Nov 22;15(12):1502. doi: 10.3390/genes15121502 (PMC11675613; doi:10.3390/genes15121502)
Supplement: Supplementary file 1 [file genes-15-01502-s001.zip › genes-3281174-supplementary.pdf]

Supplementary Table S1. Differentially upregulated genes in the datasets

Datasets	total	elements (Gene IDs)
GSE66842-up regulated GSE84587- up regulated	1	3627
GSE84587- up regulated	264	4143 127 57530 3514 57126 3827 283298 6535 22809 259 25837 2538 440556 3273 1109 4680 92292 1550 3294 8743 2161 5444 57733 2160 126969 350 5167 55937 2203 135932 344838 10050 94241 79602 4485 122622 3697 732 9154 6906 53345 6653 348 113791 2328 124 3992 9415 1565 1558 10864 8858 3158 164656 8630 26999 3026 388 7348 5053 83729 79799 199920 335 91862 64108 64284 5313 1555 3418 637 51302 59286 388646 135656 83758 2705 999 8564 6554 11254 3434 2628 25984 415 6580 200931 8875 387742 7069 2330 341 7032 3664 51471 26998 6718 386677 51181 114571 5624 3248 100526833 312 6947 229 7364 866 3080 4547 7363 26835 7033 55532 1733 1 9965 2153 79660 7103 134526 1036 2568 2690 5340 3485 57402 2165 51733 3570 55825 442038 2822 2159 2263 4359 353135 80168 57678 10991 59272 2155 1593 2528 256394 3053 10599 51299 64711 18 116842 10013 125 4072 6913 7448 1564 1610 7365 9963 220001 406991 283600 1556 197 3626 6699 3638 84962 125488 432 55620 51268 55908 5652 6360 642475 80086 6319 28970 4938 64577 590 5267 345 319 6558 84647 735 256764 80833 346606 7108 387775 8547 5266 126 7031 91543 8529 23498 283460 55349 1559 5009 392636 383 7036 55277 112399 462 5169 6898 9027 1510 55089 5919 54346 2542 9032 10071 173 7429 1369 54363 29958 7276 5207 3818 27329 10551 26007 635 7018 570 58985 148738 83733 51473 3950 1361 8710 3698 8843 5046 23491 9603 441490 130 1373 389643 55753 345275 3990 387601 257313 54967 2329 64241 119467 2056 54762 51179
GSE66842- up regulated	33	64855 7873 122416 3725 56666 55700 6809 1396 79567 6286 6303 3710 2919 51191 402 11074 23208 6867 3399 2318 6374 3576 58528 9246 1052 9636 2537 3397 27063 9235 6364 57817 5866

Supplementary Table S2. Differentially downregulated genes in the datasets

Datasets	total	Elements (Gene IDs)
GSE66842-down regulated GSE84587-down regulated	2	10590 3017
GSE84587-down regulated	600	8434 3037 90865 113763 5366 196441 57600 9736 55243 22836 51474 2825 23233 389136 79971 4060 8546 11169 253827 79652 4017 1303 5831 10401 6541 23164 9768 132789 3487 160418 11213 51715 11230 6091 120103 83464 27241 1293 54829 92745 85460 29967 7345 8682 84343 10112 4860 4651 10203 7045 387758 3554 8202 9180 6790 1000 151963 10466 9824 1728 4360 9232 90355 22974 4921 586 10560 4097 9918 5954 1012 4323 64859 55752 5874 57514 4211 1536 4015 2191 1292 9448 4144 22795 57584 28996 131965 558 4317 3912 5066 10272 5937 57333 254228 9173 56999 9859 115908 26586 23362 7042 55055 4897 27286 9404 25932 1075 5806 308 5742 6280 5968 200916 3945 7424 9509 4811 4920 6867 3676 51661 813 2014 83716 301

		57493 3399 7837 84648 27122 201725 3685 3098 6362 285761 493 79901 6723 22848 116496 8450 50810 55757 9315 2687 55215 2012 55230 1902 2331 6196 5136 93663 3572 3690 633 5347 80205 1604 27303 10257 259232 990 3908 2316 54443 10184 131544 64943 1809 2744 9697 84187 11167 1107 8974 59345 8754 3838 286148 7443 5587 4175 84959 5069 1111 2072 29127 5217 2202 253461 11183 7076 3480 323 1910 4363 4907 60681 347902 7130 3915 84135 9068 8840 8439 7025 8342 283208 483 2882 9133 4311 25800 10602 1284 85395 4256 85004 400410 51313 3553 2022 11117 8406 9843 10493 57381 2997 84188 6591 134430 91147 7077 348235 5538 114897 5068 9631 11238 83700 54431 5743 10993 3082 6482 54532 293 51665 54510 84641 54495 29940 64151 8323 7037 8916 26278 6678 166336 699 23595 960 2650 10516 1038 5329 5591 7171 3678 8038 10457 22943 7070 57045 2200 81831 5311 84668 27101 6510 781 3428 11080 23511 3015 1410 64855 5578 891 4942 5327 8898 6574 730 55839 8365 7227 1543 22873 143686 8853 1490 261729 1836 994 5360 55454 8910 10026 160428 55872 4288 23271 55742 78989 91624 10082 23704 147372 6747 7298 10512 92126 8829 23111 26115 642 5784 6617 5965 9902 241 9493 23213 1281 493869 7057 89796 23657 3675 11010 4313 56944 1503 894 8099 10085 104 5768 23404 412 11098 57182 5376 91607 8476 84168 1513 93380 83468 4548 3151 2058 24137 10797 1462 3488 9688 83448 162632 8354 79827 1634 5321 2042 2791 9787 9462 8826 55212 5793 5156 5139 2317 3490 890 440 1289 9805 1282 7267 871 5983 79188 1277 22883 7402 867 27115 23431 79801 7728 11260 6793 131566 285203 2534 2539 9497 10234 256691 10252 5118 126393 57088 2491 2059 54908 196051 55186 9793 5019 284119 2530 9055 55332 57153 84709 8531 10253 84197 89795 10051 26509 29968 7058 5783 140609 84233 81792 3832 153642 151887 100128252 3059 3569 84898 283209 472 54892 345651 9833 10778 4052 9510 5967 64682 57568 63892 1808 54680 6935 231 159195 57181 79689 51026 1545 6876 10920 7980 51520 285636 56261 397 857 25902 4919 9262 1909 4314 9358 4239 3009 7412 54552 7292 619383 50515 771 134957 10652 22856 5997 26227 5055 5010 51514 6696 80380 64175 284371 1291 7043 8204 219854 11215 55704 26585 6515 220885 25938 26872 5286 51077 10631 11031 26064 93627 60312 9945 55870 57415 57214 5395 22808 5732 65244 144811 7474 23243 3376 285527 79649 1889 2149 25963 51132 118429 2697 1290 7941 90993 7453 84515 55127 25891 8237 3977 143888 5723 23043 55276 6783 53346 5552 55183 6695 1009 5305 63895 26271 7153 79707 4883 10186 8614 79070 59 26762 9181 115123 6387 6782 143458 55033 7040 84674 7169 51232 2247 7003 152579 22955 9749 4131 150684 91663 23287 114882 8140 55075 80310 2669 28316 196 91775
GSE66842-down regulated	55	127 8349 5345 4051 350 8337 2052 3156 124 4496 10864 2194 6694 7032 39 114571 7033 3157 5264 1622 723790 4047 3638 2938 7108 3422 5005 326625 4494 27329 1361 253012 3949 57733 6307 2222 6713 1717 1915 10682 8334 3263 3053 1595 7448 100507203 6319 1551 27141 2224 383 51703 4597 763 3698
